# Telehealth Adaptation for Multidisciplinary Colorectal Cancer Clinic During the COVID-19 Pandemic

**DOI:** 10.7759/cureus.17848

**Published:** 2021-09-09

**Authors:** Blessing O Aghedo, Shane Svoboda, Leslie Holmes, Lillian Man, Yin Wu, Jeanette Linder, Christopher D'Adamo, Arun Mavanur, Kathryn Poehler, Deanna Codling, Joshua H Wolf

**Affiliations:** 1 Surgery, Sinai Hospital of Baltimore, Baltimore, USA; 2 Surgery, American University of Barbados, School of Medicine, Saint Michael, BRB; 3 Radiation Oncology, Sinai Hospital of Baltimore, Baltimore, USA; 4 Oncology, Sinai Hospital of Baltimore, Baltimore, USA; 5 Family and Community Medicine, University of Maryland Medical Center, Baltimore, USA; 6 Surgery, Johns Hopkins, Baltimore, USA; 7 Surgery, George Washington University School of Medicine and Health Sciences, Baltimore, USA

**Keywords:** colorectal cancer, rectal cancer, multidisciplinary clinic, telehealth, covid-19

## Abstract

Background

The study objectives were to transition in-person colorectal cancer multidisciplinary clinic (MDC) to a telehealth MDC (tele-MDC) format and to assess early outcomes.

Methods

A colorectal tele-MDC was devised, in which patients used remote-access technology while supervised by a clinician. The team consisted of surgeons, medical oncologists, radiation oncologists, radiologists, and pathologists. Outcomes were assessed with patient and provider surveys, using a 5-point Likert scale (higher = more favorable).

Results

A total of 18 patients participated in the tele-MDC. Surveyed patients (n=18) and physicians (n=19) were satisfied with the quality of care (mean Likert = 4.93, 4.53, respectively), and low standard deviations (range 0-1.03) across all questions reflected homogeneity in satisfaction with the metrics surveyed.

Conclusions

This pilot study demonstrates that a functional colorectal cancer tele-MDC is a feasible alternative to in-person MDC during the coronavirus disease 2019 (COVID-19) pandemic, with the potential for a high degree of patient and physician satisfaction.

## Introduction

For a patient with a new diagnosis of rectal cancer, navigating the modern healthcare system through all of the required appointments can be an overwhelming task. Patients are expected to undergo multiple imaging studies to complete the staging workup, and then meet with multiple physicians from different specialties in order to begin the appropriate treatment plan. Since locally advanced rectal cancer is typically treated with neoadjuvant chemoradiotherapy before surgical resection, the list of specialty appointments includes a minimum of three encounters (surgery, medical oncology, radiation oncology), and often others are needed as well for comprehensive care (genetic counseling, interventional radiology, enterostomal therapy). This pathway can lead to poor compliance and healthcare disparities since it can be particularly burdensome for patients with lower health literacy, limited expenses for travel, or inability to take off time from work. Recent work has also shown that “fragmented” rectal cancer care across multiple institutions can lead to delays in treatment [1].

Patient evaluation by a multidisciplinary team (MDT) for colorectal cancer consolidates care within a single group of clinicians, who work together to formulate an evidence-based treatment plan. Though there is some controversy regarding whether MDT for rectal cancer leads to improved oncologic outcomes, it is generally accepted that it leads to improved short-term metrics related to coordination of care [2]. It is no surprise, therefore, that MDT is considered to be one of the standards set by the American College of Surgeons National Accreditation Program for Rectal Cancer (NAPRC), and is mandatory for the development of a comprehensive rectal cancer program [3]. The NAPRC guideline does not require that the patient participate directly in the MDT per se, but others have described the advantages of a multidisciplinary clinic (MDC), which combines the benefits of MDT discussion with a patient encounter involving multiple specialists [4]. This approach improves the patient experience by reducing the burden of multiple clinic visits and leading to better communication between the clinical team and the patient. A comprehensive multidisciplinary plan of care is created after a single visit with input from all specialties. The patient understands the next steps in their treatment and the long-term cancer care plan without the risk of conflicting opinions that can occur when specialties are seen individually.

The coronavirus disease 2019 (COVID-19) pandemic has led to challenges for both patients and physicians in achieving timely treatments for cancer, exacerbating the aforementioned baseline difficulties. Among these, policies at the governmental and institutional levels aimed at limiting the spread of the virus have created new barriers to the traditional MDC format. Face-to-face discussion between a group of specialists and the patient, the central tenet of MDC, is not possible under pandemic restrictions because it would require a physical gathering. Patients may also be rightly apprehensive about participating in discussions in-person with a large group. The alternative to MDC, which would involve separate sequential clinic visits, would only increase the risk of patient exposure to the virus by requiring multiple trips to a healthcare facility.

As more and more of the healthcare industry moved to a virtual format to circumvent disruptions in patient care, the hypothesis in this study was that colorectal MDC could be successfully transitioned to a telehealth platform. While remote physician-patient encounters have emerged as a new standard, telehealth adaptations of colorectal cancer MDC have not yet been described. The objectives of this pilot study were to transition in-person MDC to a telehealth MDC (tele-MDC) format and to assess early outcomes for patient and physician satisfaction. The format that is described in this report includes tele-conferencing for the MDT discussion, and consolidation of multiple physician visits into a single supervised telehealth encounter in the clinic.

This article was previously presented as a meeting abstract at the 2021 ASCRS (American Society of Colon and Rectal Surgeons) Annual Scientific Meeting on April 24, 2021.

## Materials and methods

Study design

This study was a single-institution pilot study that began in April 2020 after restrictions due to the COVID-19 pandemic which halted the in-person MDC. The study was exempt by the Institutional Review Board based on applicable federal regulations (45 CFR 46).

MDC clinic development

A tele-MDC was devised, in which patients with colon, rectal or anal cancers could participate in a clinic appointment with multiple specialists simultaneously using remote-access technology, while remaining compliant with pandemic restrictions. In terms of administrative personnel and clinical staff, the clinic was a natural outgrowth of the existing in-person MDC that had been operational for approximately one year pre-pandemic. Referrals were coordinated by the office administrators in the Department of Surgery, and all visits were scheduled during a designated two hour weekly timeslot. Once a patient was referred, the case was reviewed by a physician assistant (PA) and/or surgeon and the appropriate staging workup was coordinated for the patient’s cancer type, according to guidelines from the National Comprehensive Cancer Network (NCCN). Requisite staging studies were completed prior to tele-MDC appointment. If the patient’s pathology slides had been developed and read at an outside institution, they were obtained and reviewed in-house.

Team composition

The clinical team was modeled after the NAPRC standard 1.2, and was comprised of colorectal surgeons, PAs, medical oncologists, radiation oncologists, a radiologist, and a pathologist [3]. A clinical nutritionist was part of the MDT during the early experience until this individual was needed in other capacities as part of pandemic contingency planning at the institution. A genetic counselor was invited to participate if relevant. Primary care providers and gastroenterologists were invited to attend on a case-by-case basis.

Clinic workflow

**Figure 1:**
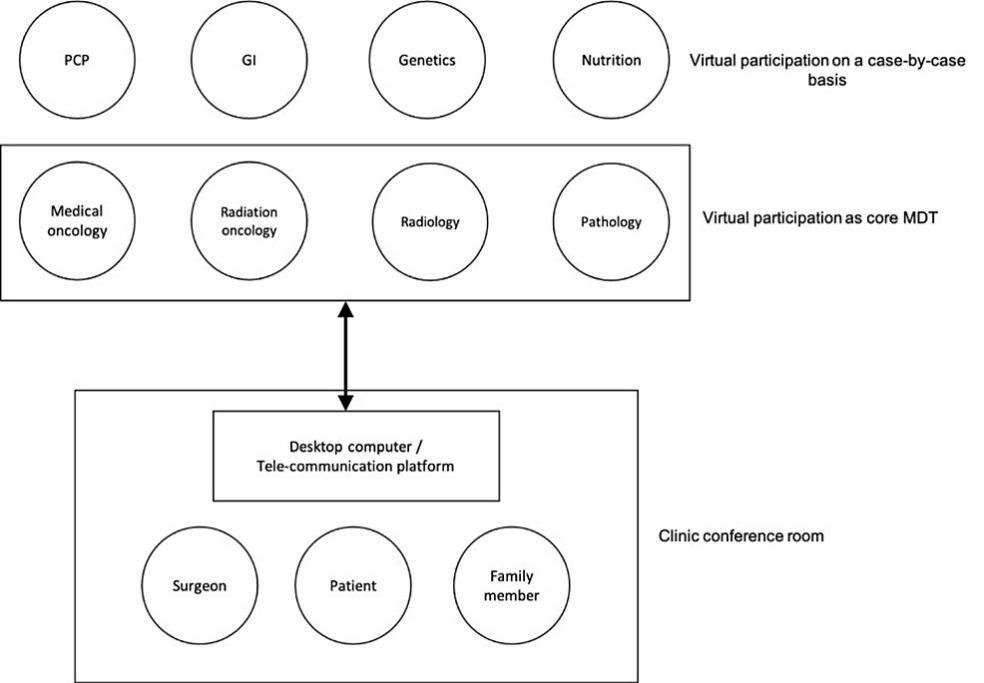
Schematic representation of the tele-MDC team The format that was selected for tele-MDC was a “guided” telemedicine encounter. The patient was brought to clinic where the surgeon assisted the patient in navigating a remote encounter with multiple specialists. This removed the technological burden of telemedicine from the patient and family, and allowed them to focus fully on engaging the providers. The remote-access format also allowed for other participants not traditionally included in MDC, namely, the patient’s primary care provider (PCP), gastroenterologist (GI), a clinical nutritionist and a genetic counselor.

Each tele-MDC session began with a virtual case conference, in which clinical data were reviewed on a remote platform by the MDT, and a preliminary plan for further diagnostic workup and/or therapy was devised. Patients were then brought to the clinic conference room in person where, with direct guidance from the surgeon, they were introduced to the other specialists in the virtual platform, using both video and audio communication. This format was chosen to ensure the patient would not have difficulties with the technology, to establish rapport in person with a team representative given the sensitive nature of the discussion, and to allow for a physical examination by the surgeon (Figure 1).

Each specialist was given time to interview the patient and discuss the details of their role in the treatment plan. The surgeon performed the physical examination, and this was intentionally not done in view of the remote tele-communication setup, to assuage potential concerns about privacy during this portion of the encounter. Physical exam findings were reported to the group following the examination. In some cases, the tele-MDC appointment occurred after an initial visit with the surgeon, in which a physical examination had already been performed. The exam was not repeated in tele-MDC for these patients. The final comprehensive treatment plan was discussed with the patient and any family members in attendance and all questions were answered.

Security of electronic health information

All virtual appointments utilized Zoom, through an institutional platform with increased cybersecurity from the commercially available application. MDC providers, including those in the clinic with the patient, used a link created on this secure platform to connect to the conference and the patient encounter.

Billing for tele-MDC

Prior to each tele-MDC visit the patient’s insurance was verified and reviewed by a designated clinic medical assistant (MA). The MA assessed whether or not the patient’s insurance required a separate referral for each specialty. If a referral was not needed, then the patient was able to come in and see each of the providers virtually without delay. The primary surgeon performing the exam will bill for an in-person office visit. The providers that consult virtually bill for virtual visits. If the patient’s insurance required a referral, the clinic MA reached out to the primary care provider and asked for individual referrals for each provider (medical oncology, radiation oncology, and colorectal surgery).

Data collection and analysis

Outcomes were assessed with the patient and provider surveys, each comprised of questions using a 5-point Likert scale (with higher scores indicating more favorable outcomes). The surveys were based on previously published instruments for assessment of tele-medicine encounters, with modifications in order to capture the desired study outcomes [5,6]. Physician and patient surveys were collected after each clinic and reviewed in aggregate for the entire study interval. The tele-MDC underwent several preliminary sessions while the logistics were finalized, and therefore four patients in the early experience are not represented in the survey data. Descriptive statistics were computed in SAS version 9.4.1 (SAS Institute Inc, Cary, NC).

## Results

A total of 18 patients have been evaluated at the tele-MDC since its inception at the onset of the COVID-19 pandemic (Table 1). This cohort included patients with diagnoses of colon (11.1%), rectal (77.7%), and anal (5.5%) cancer, and a single case of recurrent uroepithelial cancer. All patients were referred to the clinic due to the need for a multidisciplinary treatment plan. Average time between tele-MDC treatment and initiation of definitive therapy was 30.9 days (SD 13.1). This included three patients with obstruction, who underwent pre-treatment laparoscopic diverting colostomy formation and two weeks of postoperative recovery prior to initiation of treatment.

**Table 1:** Characteristics of the tele-multidisciplinary clinic (MDC) cohort ^a^Numbers in parentheses represent standard deviation for continuous variables and frequency for categorical variables. ^b^There was a single case of recurrent uroepithelial carcinoma

	tele-MDC Cohort
N	18
Age, years	64.9 (14.1)^b^
Gender	
Male	12 (66.6)
Female	6 (33.3)
Diagnosis	
Colon cancer	2 (11.1)
Rectal cancer	14 (77.7)
Anal cancer	1 (5.5)
Other^b^	1 (5.5)
Cancer stage	
I	0
II	7 (38.9)
III	8 (44.4)
IV	3 (16.7)
Time of tele-MDC to treatment, days	30.9 (13.6)

Scores from 19 surveyed physicians reflected overall satisfaction with the clinic format (Table 2). Physician satisfaction with the technological aspects of the clinic (mean score Question 1 = 4.32, Question 2a, 2b = 4.32, 4.21 respectively) reflects the rarity of technological difficulties interfering with the patient-physician interaction during the course of this pilot. There was a low level of concern among providers that the virtual format would lead to medical errors (Question 4 = 2.05). In general, physicians were satisfied with teamwork, communication, and quality of care (Questions 7, 8, 9= 4.68, 4.68, 4.53 respectively).

**Table 2:** Physician survey results from the pilot tele-multidisciplinary clinic (MDC)ᵃ ^a^ Results were compiled from 19 completed physician surveys. ^b^ Numbers represent mean 5-point Likert scale values, with the exception of question 4, higher scores indicating more favorable outcomes. Numbers in parentheses are standard deviations.

Question	Survey Text	Mean score
1	It was easy to use the virtual clinic technology.	4.32 (0.58)^b^
2	I was satisfied with	4.32 (0.58)
	a. the video quality of this visit.	
	b. the audio quality of this visit.	4.21 (0.63)
3	I was confident in the privacy and security of patients’ health data while using the virtual clinic.	4.26 (0.65)
4	I had concerns regarding making medical errors that would be avoided if an in-person clinic was done.	2.05 (1.03)
5	Compared to an in-person clinic, this virtual multidisciplinary clinic was:	4.05 (0.91)
	a. time-saving.	
	b. cost-saving	3.79 (0.79)
6	I had better recommendations in the treatment plan due to input from other disciplines.	4 (0.77)
7	I was satisfied with the teamwork of the healthcare team	4.68 (0.48)
8	There was clear communication between the members of the multidisciplinary team.	4.68 (0.48)
9	I was satisfied with the quality of care provided in this visit.	4.53 (0.51)
10	I am open to incorporating a virtual multidisciplinary clinic in my practice.	4.42 (0.69)

Patient survey results similarly reflected a high degree of satisfaction with the clinic (Table 3). In particular, patients felt that the virtual technology was easy to use (Question 1 = 5.00). Patients gave high ratings for the audio and video quality of the visit (Questions 2, 3; 4.93, 4.93 respectively), the quality of communication (Questions 7, 8, 9= 4.93, 4.93, 4.93 respectively), and generally agreed that they would recommend the virtual clinic to other patients (Question 12 = 4.93). The standard deviation of the satisfaction scores among patients and physicians was low (SD < 1.04 for all survey questions).

**Table 3:** Patient survey results from the pilot tele-multidisciplinary clinic (MDC)ᵃ ^a^Results were compiled from 18 completed patient surveys. ^b^Numbers represent mean 5-point Likert scale values, with higher scores indicating more favorable outcomes. Numbers in parentheses are standard deviations.

Question	Survey Text	Mean score
1	The virtual technology was easy to use.	5.00 (0)^b^
2	I was satisfied with the:	4.93 (0.27)
	a. Audio quality of this visit.	
	b. Video quality of this visit	4.93 (0.27)
3	I was confident in the security and privacy of my personal health information while using the virtual clinic.	4.79 (0.58)
4	Compared to an in-person clinic, this virtual multidisciplinary team clinic visit was time-saving.	4.86 (0.36)
5	I was satisfied with the time spent with my physicians during this visit.	4.86 (0.36)
6	I liked the cooperative effort of the healthcare team involved in my treatment plan.	4.93 (0.27)
7	I was involved in the discussion of my care plan.	4.93 (0.27)
8	My health concerns for which the appointment was made were addressed.	4.93 (0.27)
9	My care plan was explained to me in a clear manner.	4.93 (0.27)
10	Overall, I was satisfied with the quality of care received in this visit.	4.93 (0.27)
11	I would like to have another virtual consultation in the future.	4.93 (0.27)
12	I would recommend this virtual multidisciplinary clinic to other patients.	4.93 (0.27)

## Discussion

This pilot study demonstrates that tele-MDC is a feasible alternative to in-person MDC during the COVID-19 pandemic, with the potential for a high degree of patient and physician satisfaction. In a time of relatively limited healthcare access for cancer patients due to both institutional and governmental regulations, tele-MDC was a viable option for timely, comprehensive cancer care while remaining compliant with COVID-19 restrictions. The virtual format was well received, with low standard deviations across all satisfaction scores reflecting relative homogeneity in satisfaction with the tele-MDC program among both patients and physicians. This is to our knowledge the first description of a virtual MDC adaptation for colorectal cancer patients.

Interestingly, despite the fact that the tele-MDC was designed as a contingency in response to pandemic restrictions, there were certain features that emerged as advantageous over the pre-pandemic format. From the physician perspective, remote technology eliminates the need for travel and allows more consistent and punctual participation, since not all team members are located in the same part of the medical center. Some potential logistic barriers to in-person conferencing are removed. For example, there is no need for a reserved conference space, and no need for technical accommodations for special imaging software, since clinical images (radiology and pathology) could be shared from the specialist’s own office workstation. From the patient perspective, tele-MDC can allow participation of close contacts who would otherwise be excluded from the encounter, such as the primary care physician, or remote family members. Because tele-MDC is easily accessible to patients who are unable to travel to multiple appointments due to associated costs (travel expenses, time off of work, etc), it also has the potential to reduce disparities in cancer care due to socioeconomic status. These potential advantages may make certain elements of tele-MDC attractive additions to the traditional format even after the COVID-19 pandemic subsides.

There were several lessons learned while developing the tele-MDC at this institution. First, it was important to have a pre-existing model for MDC that was fully functional prior to the transition to tele-MDC. This ensured that all stakeholders had already allocated sufficient resources, specifically in terms of staffing and time. Though satisfaction surveys are not available from the institution’s in-person clinic prior to the pandemic, it was well-attended by clinical team-members and subjectively was well received by patients. The adaptation to a remote format was therefore a shared vision that appealed to all parties involved. Second, because the format for the tele-MDC was new to patients and family members, it was helpful to provide an introduction to the tele-MDC arrangements prior to the appointment in order to set proper expectations. This was typically done by phone when the visit was being arranged and then reinforced with a brief discussion before entering the conference room during the visit. Third, toward the middle of the pilot, a provider stationed at a clinical workstation was added remotely to the tele-MDC discussion. The job of this team member was to place any necessary orders and complete a summary worksheet, which was provided to the patient at the time of departure in a folder. This helped reinforce the MDC plan with visual aids and references, and helped with immediate scheduling of any recommended follow-up testing.

The COVID-19 pandemic has forced a shift to virtual format across the full spectrum of healthcare services, and others have described telehealth adaptations for MDT or MDC previously for other clinical contexts aside from colorectal cancer [7-11]. In what is the closest example to the work in this study, Grenda et al. reported how the Multidisciplinary Lung Cancer Clinic at the University of Pennsylvania was adapted to a telehealth format [8]. In this model, patients are seen via remote encounter by each specialist in turn, without an in-person evaluation. This differs from the format chosen in this pilot, which permitted a single physician to interact with the patient directly in the clinic and perform a physical examination. A single physician contact was deemed necessary for colorectal tele-MDC for several reasons. First, it obviated the patient from having to deal with any technological issues, or anything at all other than the content of the discussion. This was especially helpful for older patients, who in general were less adept at using the technology. Of additional importance, by allowing the patient to interact with the surgeon directly, it was possible to include data from the physical examination in the final plan. The information gained from the digital rectal examination is essential in the treatment of rectal cancer (determination of the lesion’s precise location, clinical staging, and sphincter function). Unlike the case for other tumors, including lung, in which direct physical examination of the tumor itself is not possible, MDC for rectal cancer without a physical examination would rely on incomplete data to produce a recommendation. The present pilot also differed from the MDC described by Grenda et al. in that specialists spoke to the patient altogether, rather than in sequence [8]. A simultaneous encounter was chosen due to the nature of multi-modal therapy for rectal and anal cancers. Patients often had questions pertaining to multiple specialists which could be answered as a team, better ensuring unified messaging and patient comprehension.

Others have used survey data to assess the satisfaction of participants in virtual MDT. Rajasekaran et al. described an adaptation of a sarcoma MDT at the University of Oxford that was also in response to the COVID-19 pandemic [12]. In this study, 75% of the participants felt the virtual MDT was equivalent to in-person discussion and 55% wished to continue the virtual format after easing of pandemic restrictions. The data in the current study are more uniformly favorable with respect to these questions. Aghdam et al. published a review of telehealth in MDT months before the COVID-19 pandemic began, and therefore, many more recent publications were obviously not included [13]. The authors of this review highlighted research and innovation across many specialties including dermatology, cardiology, neurology, oncology, and palliative medicine. They also described the potential for collaboration between hospitals in constructing a virtual MDT, to bring together a group of clinicians across a wide geographic area. This was not an option that was pursued in the current study, but one that certainly may be considered as the tele-MDC continues to grow in experience.

Conclusions from this study are limited by a small cohort size and the potential for response bias within the patient and physician surveys. In general, patients filled out their surveys in person before leaving clinic which reduced recall bias. Certain outcomes including the number of no-show appointments or appointment cancellations were not captured, and therefore, patient survey results may be overestimating the satisfaction of the total group of patients who made contact with the clinic. In many cases, COVID-19 restrictions and the need for testing and/or quarantine led to delays in obtaining appointments for pre-clinic or post-clinic imaging or biopsy, therefore, it is difficult to accurately assess metrics related to treatment timing, for example, time to treatment initiation. Traditional MDT has been linked to improved rectal cancer outcomes, and it has therefore been incorporated into the NAPRC standards [3,14,15], but whether or not tele-MDC can be associated with similar clinical benefits was beyond the scope of this pilot project. The relationship between tele-MDC and cancer outcomes will be useful to study as a larger cohort of patients is accumulated for this clinic.

## Conclusions

Colorectal cancer tele-MDC is a feasible option for comprehensive care of patients during the COVID-19 pandemic. The format reported included teleconferencing for the MDT discussion, and consolidation of multiple physician visits into a single supervised telehealth encounter in the clinic. This allowed access for patients that was compliant with both NAPRC standards as well as pandemic restrictions. Both patients and physician team members were satisfied with the quality of care for this pilot tele-MDC. Ease of access, reduced resource utilization, and inclusion of a broader team are all potential advantages of tele-MDC that should be considered as virtual formats integrated into post-pandemic care.
